# A novel approach to predict the arctic stratospheric ozone from stratospheric polar vortex dynamics using explainable machine learning

**DOI:** 10.1038/s41598-025-24379-9

**Published:** 2025-10-20

**Authors:** Anish Kumar, Joyjit Mandal, Sina Mehrdad, Christoph Jacobi

**Affiliations:** 1 Institute for Meteorology, Leipzig University, Leipzig, Germany; 2 Department of Computer Science, Central University of Rajasthan, Ajmer, India

**Keywords:** Stratospheric polar vortex, Arctic ozone loss, Explainable machine learning, Atmospheric science, Atmospheric dynamics

## Abstract

A significant decreasing trend of Arctic stratospheric ozone has been observed since 2019, with the first reported ozone hole in the Arctic Stratospheric Polar Vortex (SPV) in 2020, raising concerns for humanity. This underlines that it is essential to develop an algorithm capable of predicting Arctic ozone levels, preferably using minimal computing resources. This study presents a novel approach for ozone prediction based on the morphological and dynamical properties of the SPV utilizing a explainable machine learning approach. XGBoost exhibits good agreement with the observations, achieving an $R^2$ score of 0.80 and a correlation of 0.91. The algorithm accurately predicts the daily and seasonal patterns of ozone variations. It successfully captures the pattern of the lowest recorded ozone levels in 2020, though it overestimates ozone values by approximately 20 Dobson units. Moreover, in some years the predicted ozone values also show a strong alignment with the observations. Notably, the algorithm relies solely on physics based features of the SPV to predict chemical ozone loss, demonstrating the potential of dynamical parameters in predicting the ozone variability. It could serve as a tool for projecting future Arctic ozone variability by utilizing input from climate models that lack interactive chemistry.

## Introduction

Ozone acts as a shield against harmful ultraviolet (UV) radiation, preventing it from reaching the Earth’s surface. Depletion of ozone poses significant risks to public health and disrupts climate patterns^1–3^. The ozone hole was first discovered by Farman^4^ in 1985 over Antarctica during the spring. Anthropogenic ozone depleting substances (ODSs) and a strong, stable stratospheric polar vortex (SPV) are the primary drivers of ozone loss^4,5^. Studies indicate a positive trend in ozone recovery since the 1990s over Antarctica due to the Montreal Protocol, which restricted ODS emissions. Notably, 25 years have passed since ODS levels peaked^1,2,6^.

Ozone loss over the Arctic, however, remains a significant concern. The stratospheric ozone (here after ozone) hole over the Arctic is an alarming issue for humankind due to the dense human settlements in the Northern Hemisphere (NH). Time-varying 20-year trends in Arctic ozonesonde data (1994–2022) show significant positive trends for time windows ending before 2017 at some stations, no significant positive trends for windows ending after 2017, and significant negative trends at several stations for windows ending after 2019^7^. The Antarctic ozone hole, defined as a total ozone column (TOC) below 220 Dobson Units (DU), is a well documented phenomenon. However, for the first time in history, the Arctic experienced an ozone hole in 2020, lasting approximately three weeks in different regions of the SPV, which includes some days of December, January, March, and April, with extremely low TOC values in March and April^8^. Although this Arctic ozone loss was not as extensive as the one over Antarctica, Kuttippurath et al.^8^ reported TOC values below 220 DU for several weeks during the Arctic winter, suggesting that we may be entering a new era of ozone depletion^8,9^ as a transient effect that may end due to the ongoing phase-out of ODSs^1,2,6,10,11^. However, Gathen et al.^9^ warned that large Arctic ozone losses could persist or even worsen by the end of the century if greenhouse gases (GHGs) continue to increase steeply, as based on model projections. This concern is also supported by multiple studies^12–17^, which indicate that ongoing climate change driven by anthropogenic GHG emissions leads to stratospheric cooling over time. This cooling favors a stronger and more stable vortex, promoting the formation of polar stratospheric clouds (PSCs), which are closely linked to severe ozone loss.

The volume of PSCs is highly correlated with potential ozone loss^13,16,18,19^. During the polar night, hydrochloric acid (HCl) and chlorine nitrate (ClONO$_2$) are sequestered in reservoir gases, while the SPV isolates the air mass within it. If temperature drops below 196 K, PSCs form. On the surface of these PSCs, heterogeneous reactions break down reservoir species, releasing active chlorine, which then catalytically destroys ozone once sunlight returns in spring^7^. Rex et al.^13^, reported a correlation coefficient of 0.99 between TOC loss and PSC volume, based on data (1992-2003) of January, February, and March. This empirical ratio enables the investigation of the impact of stratospheric temperature changes on Arctic ozone loss. It also provides the flexibility to explore the relationship between Arctic SPV dynamics and ozone loss. In the past few decades, three Arctic winters (1996-97, 2010-11, and 2019-20) have exhibited anomalously strong SPV conditions during late winter and early spring, specifically in February, March, and April (FMA)^20–22^. Hereafter, anomalously strong Arctic SPV events will be referred to as Exceptionally Strong Vortex (ESV). ESVs are associated with significant ozone loss; for example, during the 2019–20 winter the Arctic recorded the lowest ozone level on record, leading to a localized ozone hole^8,21^.

Considering the direct relationship between TOC loss and SPV dynamics, statistical models can be utilized to investigate underlying patterns. These models are computationally less expensive and do not require complex mathematical equations compared to climate models. Several studies have attempted to predict Arctic SPV variability using Machine Learning (ML) and Deep Learning (DL) models^23–29^. These studies primarily focus on capturing sudden stratospheric warming (SSW), which is a frequent extreme event occurring approximately every two years in the Arctic SPV^30^. However, another extreme Arctic SPV condition, namely ESV during FMA should also be considered. ESV is associated with ozone loss^21^, yet no study has specifically addressed this phenomenon over the Arctic using ML. Although some studies have attempted to model ozone using ML/DL approaches^31–33^, they did not specifically focus on Arctic ozone loss. Moreover Nowack et al.^31^, predicted global ozone distribution based on temperature, but the training and testing data were entirely derived from climate models.

Addressing the research gap in Arctic ESV and its association with ozone loss during late winter and early spring in the field of ML, we present, to the best of our knowledge, the first ML based algorithm designed to predict ozone loss. This algorithm utilizes the dynamical and morphological properties of the Arctic SPV during FMA. The input features of the ML algorithm are derived from the physical properties of the Arctic SPV, with a specific focus on detecting Arctic ozone loss. The algorithm is developed based on the previously reported relationship between Arctic stratospheric ESV properties and ozone loss^20–22^. It is trained and tested on observational and reanalysis data and can be integrated into any bias-corrected climate model, e.g., from the Coupled Model Intercomparison Project (CMIP) to project future Arctic ozone loss. Given that Arctic ozone depletion may persist or even worsen by the end of the century^9^, our approach may provide a valuable tool for interpreting future climate projections.

**Fig. 1:**
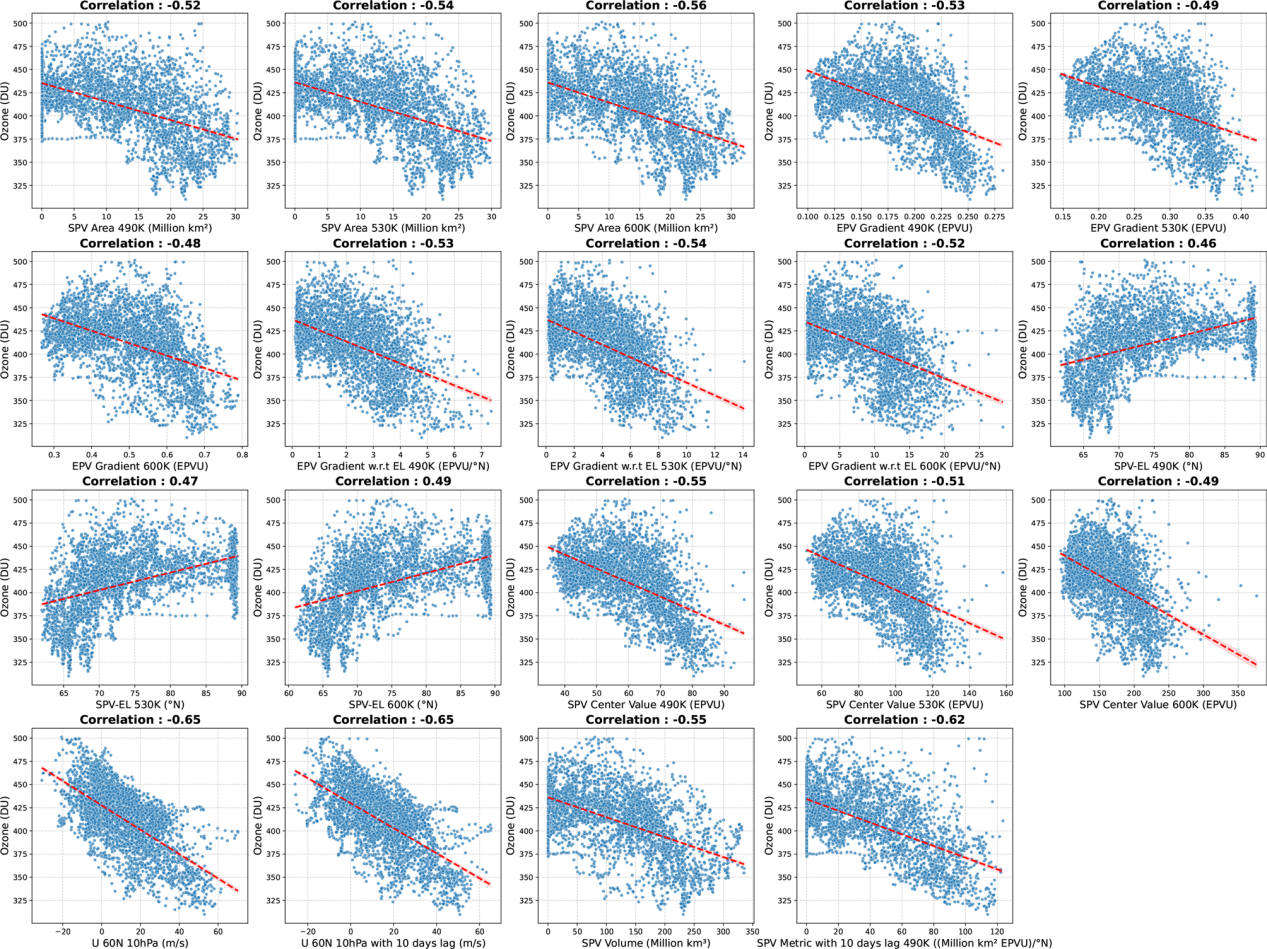
Scatter plots of polar cap TOC vs. each parameter used in the algorithm. Correlation coefficients are provided in the respective panels.

**Fig. 2:**
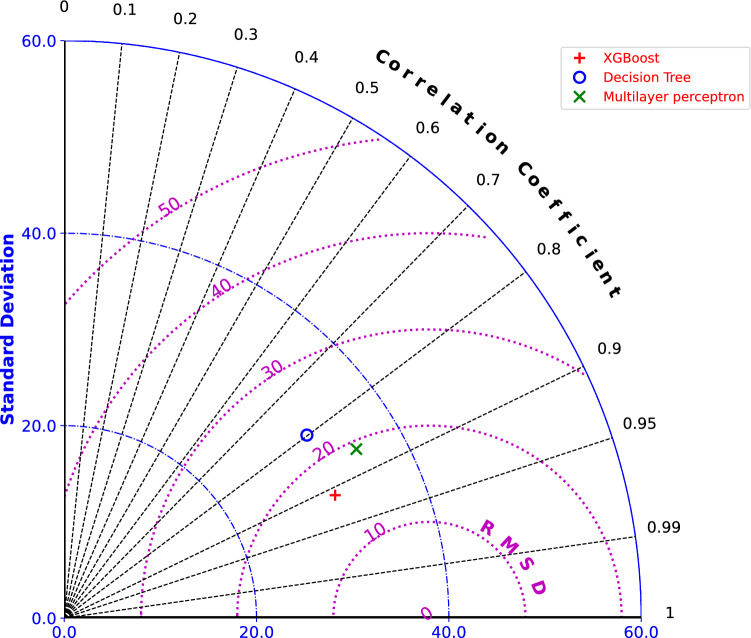
Comparison of different ML algorithms based on the mean of 10 different random seeding values, using Taylor diagram, representation of SD, correlation coefficient and RMSD.

## Results

### Comparisons of different machine learning algorithms

To develop the optimal algorithm for predicting ozone, three different ML models were tested, namely XGBoost^34^, Decision Tree (DT)^35^, and Multilayer Perceptron (MLP)^36^. Figure 1 illustrates the correlation between the algorithm’s input features and ozone; please refer to the Methods section for details. Figure 2 presents a comprehensive statistical summary of the performance of three ML models based on the mean of ten different random seeding values, comparing their performance in terms of standard deviation (SD), correlation coefficient (R), and root mean square deviation (RMSD) against ozone observations. Table 1 shows the robustness of the comparison by providing the mean and SD over ten different random seeding values. Among the three models, XGBoost performed best, achieving the highest $R^2$ (coefficient of determination) score of 0.80, explaining 80% of the variance in observations. It also had the lowest root mean square error (RMSE) of 16.78 and mean absolute error (MAE) of 13.01, indicating precise and minimally error prone predictions of ozone. Additionally, a correlation of 0.91 demonstrated the strongest agreement with the observations. Notably, the SD of random seeding for all metrics was the lowest among all ML algorithms, highlighting its high stability and consistency across different runs, further strengthening its reliability (Table 1). DT was the weakest model in this comparison, with the lowest $R^2$ score (0.64), the highest RMSE (22.61) and MAE (18.01), a lower correlation (0.82), and greater sensitivity to random initialization compared to the other two models. MLP performed slightly better than DT but was significantly outperformed by XGBoost, with an $R^2$ score of 0.65, an RMSE of 22.43, an MAE of 17.52, and a correlation of 0.85. While MLP exhibited moderate stability in terms of randomness, it showed greater variability compared to XGBoost.

The Taylor diagram (Fig. 2) further validates the performance ranking in the same order, XGBoost is closest to the reference point, with the highest correlation, the lowest RMSD, and an SD close to the reference. The reference point serves as the benchmark, indicating perfect correlation and zero SD. DT is the farthest from the reference point, while MLP falls between XGBoost and DT. Therefore, XGBoost is the most suitable ML model for developing our algorithm to predict ozone based on the morphological and dynamical properties of the SPV, as it demonstrates high precision, stability, and consistency across multiple runs. Comparisons of different ML models using various parameters such as interannual variability of ozone standardized anomalies, frequency distribution of observed and predicted ozone values, scattered plot of observed and predicted ozone values, and time series of observed and predicted ozone levels for the FMA months from 2016 to 2024 (Testing period of algorithm) are provided in supplementary file.

**Table 1:** Different ML algorithm performance metrics, based on the mean and SD of ten different random seeding values.

Model	R^2^ Score	RMSE	Correlation	MAE
XGBoost	0.80 (± 0.00)	16.78 (± 0.10)	0.91 (± 0.00)	13.01 (± 0.09)
Decision Tree	0.64 (± 0.05)	22.61 (± 1.62)	0.82 (± 0.03)	18.01 (± 1.41)
Multilayer Perceptron	0.65 (± 0.05)	22.43 (± 1.49)	0.85 (± 0.02)	17.52 (± 1.05)

**Fig. 3:**
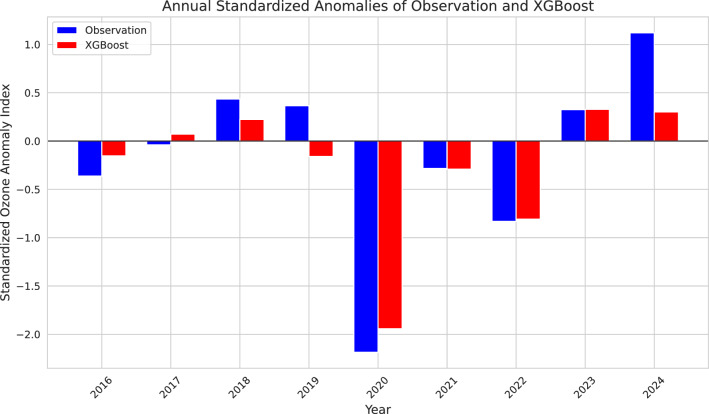
Annual standardized anomaly variations of observation and XGBoost Algorithm.

**Fig. 4:**
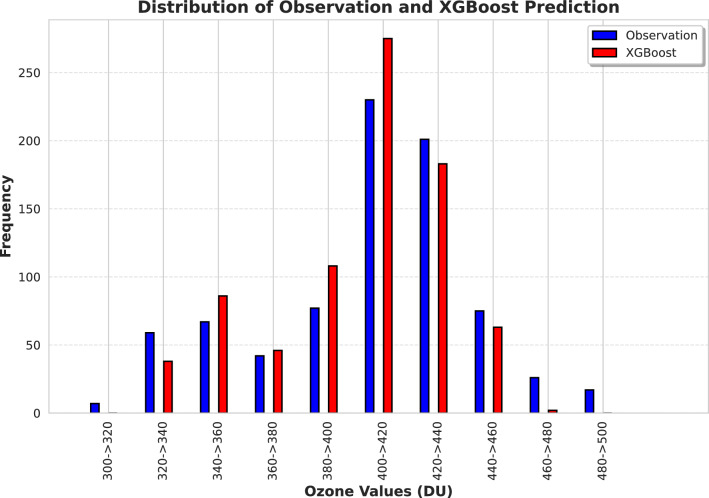
Ozone distribution of observation and XGBoost prediction.

### Performance of XGBoost algorithm

XGBoost performed the best among the other ML models; therefore, in this section, we provide a detailed overview of the XGBoost‘s performance against ozone observations. Figure 3 illustrates the interannual variability of ozone standardized anomalies. Standardized anomalies ensure that variations are normalized, allowing for a clearer comparison of relative changes rather than absolute differences in ozone concentrations. The general pattern of anomalies is well represented by the algorithm, which follows the trends of observed standardized anomalies, and captures negative and positive anomaly indices, indicating that the algorithm aligns with interannual ozone variability. Fluctuations in both observations and predictions suggest the presence of external influencing factors such as stratospheric dynamics, climate variability, and chemical processes affecting ozone levels. A close representation of the anomaly index is observed in 2021, 2022, and 2023. However, minor deviations in amplitude are noticeable in 2016, 2017, 2018, and 2020, while major deviations are reported in 2019 and 2024.

**Fig. 5:**
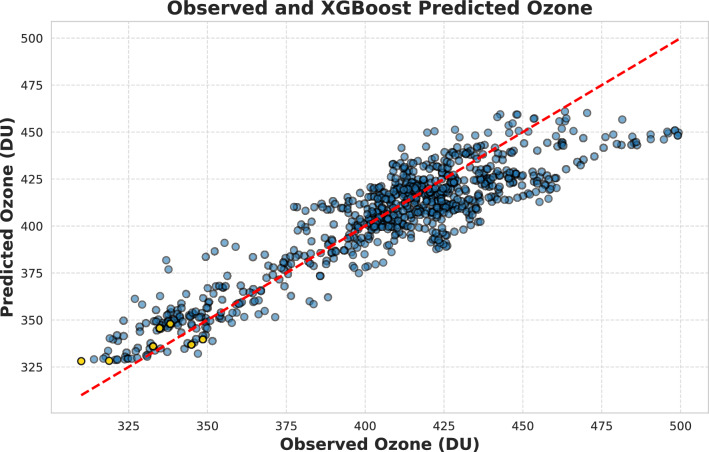
Observed and XGBoost predicted ozone with 0.91 correlation coefficient. The yellow filled symbols represent the dates when the ozone hole was recorded^8^ (2020-03-12, 2020-03-13, 2020-03-17, 2020-03-18, 2020-03-19, 2020-04-01, 2020-04-02).

Figures 4 and 5 together illustrate the agreement between observed and predicted ozone values. Figure 4 presents the frequency distribution and shows that observed and predicted distributions appear to be aligned with slight deviations, indicating that the algorithm reproduces the central tendency of ozone values. Moreover, Fig. 5 shows a correlation coefficient of 0.91, which indicates a strong positive relationship between the two. This suggests that the algorithm has high predictive accuracy. However, the model struggles to predict particularly high ozone concentrations, as indicated in Fig. 5, where higher ozone values deviate more from the reference line and Fig. 4 shows lower frequencies in the tails (both low and high ozone values), suggesting that the model may have difficulty in capturing extreme ozone values, particularly high ozone values (460–500 DU). Considering the 2020 ozone loss, the yellow dots (in Fig. 5) represent data points corresponding to days when an ozone hole was reported over the Arctic (March 12, 13, 17, 18, 19, April 1, and April 2, 2020)^8^. In particular, all yellow data points are close to the reference line in comparison to moderate ozone value (400-450 DU) prediction, with one data point being particularly very close to the reference line. This suggests that the algorithm captures ozone loss to some extent. Notably, capturing low ozone values is crucial for identifying potential threats due to ozone loss. In the next section, the 2020 extreme ozone loss event will be discussed in detail in terms of the algorithm’s performance.

**Fig. 6:**
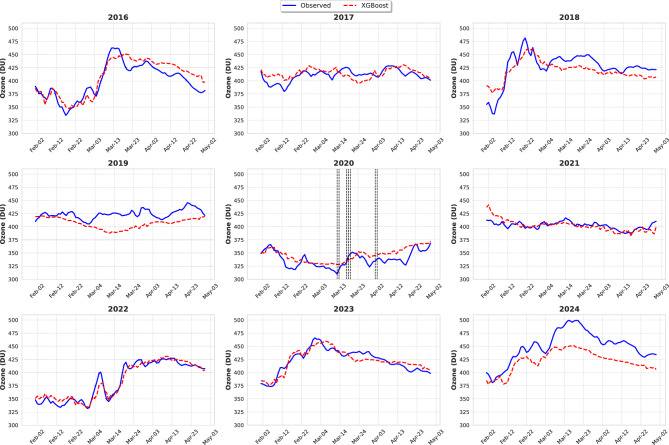
Time series of Observed and XGBoost predicted ozone during FMA for the testing period (2016-2024). Black dotted vertical lines represent dates when an ozone hole was recorded in 2020.

Figure 6 presents the time series of observed and predicted ozone levels for the FMA months from 2016 to 2024. The algorithm performed well in reproducing the general daily and seasonal trends of ozone levels. Predicted ozone values closely align with observations particularly in 2021, 2022, and 2023. In 2016, 2017, and 2018, the predicted ozone values also show a strong alignment with observations, albeit with slight over or underestimation. However, the model underestimates TOC in 2019 and 2024. In 2024, observed ozone values reached up to 500 DU. While the algorithm captured the general pattern of high ozone values, it did not fully replicate the observed peak concentrations. As the TOC reached its highest level ever over the Arctic in March 2024^37^, our algorithm lacks any training data for such conditions. In 2020, the predicted ozone values closely followed the observed pattern, with a deviation of approximately 20 DU. The dotted vertical black lines represents the days when the ozone hole was recorded. On these days, the algorithm’s predictions align well with the observed ozone values. In particular, on 17 March 2020, the observed and predicted ozone values are identical, demonstrating the effectiveness of the model to capture the loss of ozone in the NH.

The ML algorithm learns the relationship between the stratospheric dynamics and ozone variability using the given features (see Table 2). Moreover, sensitivity tests of the training years were conducted with the same testing years to verify the algorithm’s performance. In the first test, we randomly removed five years from the training period (1985–2015), repeated this process 100 times, and evaluated the model performance for each iteration. The model performance across iterations yielded mean values of $R^2$: 0.79 (±0.00), RMSE: 17.13 (±0.35), correlation: 0.90 (±0.00), and MAE: 13.30 (±0.39). In the second test, we restricted the training to an earlier period (1985–2000), deliberately excluding more recent years (2001–2015), and then tested the performance over the independent period 2016–2024. The model still reproduces the variability of ozone with reasonable skill ( $R^2$: 0.77 (±0.00), RMSE: 17.65 (±0.16), Correlation: 0.88 (±0.00), and MAE: 14.04 (±0.14)), indicating the model‘s ability to maintain similar performance even after more than a decade. This may lend some confidence that it may also be used for future scenarios.

### Arctic stratospheric ozone hole 2020

The Arctic stratospheric ozone hole was reported for the first time in 2020^8^. An ozone hole is identified when ozone values drop below 220 DU over a specific region in the stratosphere. Certain locations within the SPV recorded ozone values below 220 DU on March 12, 13, 17, 18, 19, April 1, and April 2, 2020^8^. In this study, we used ozone data averaged over the polar cap ($> 63^\circ$ N), which prevented our reported ozone values from falling below 220 DU. However, despite averaging, the polar cap ozone values recorded their lowest level ever on March 12, 2020^21^, with our algorithm overestimating the values by approximately 20 DU. However, on March 17, the predicted and observed ozone values were very close, as seen in the 2020 FMA time series (Fig. 6) and the yellow dots in the scatter plot (Fig. 5) are also close to the reference line. The ozone value distribution in Fig. 4 further confirms that while the algorithm did not exactly capture the lowest ozone values, it still provided a close approximation. Similarly, the standardized anomaly variations for 2020 (Fig. 3) show a slight difference of 0.2 units between observed and predicted values. Considering all these evaluation parameters and the alignment of the observed and predicted time series during the FMA months of 2020, we conclude that our algorithm is capable of depicting ozone loss due to ESV in the NH, as the 2020 ozone loss was the result of ESV^21^.

**Fig. 7:**
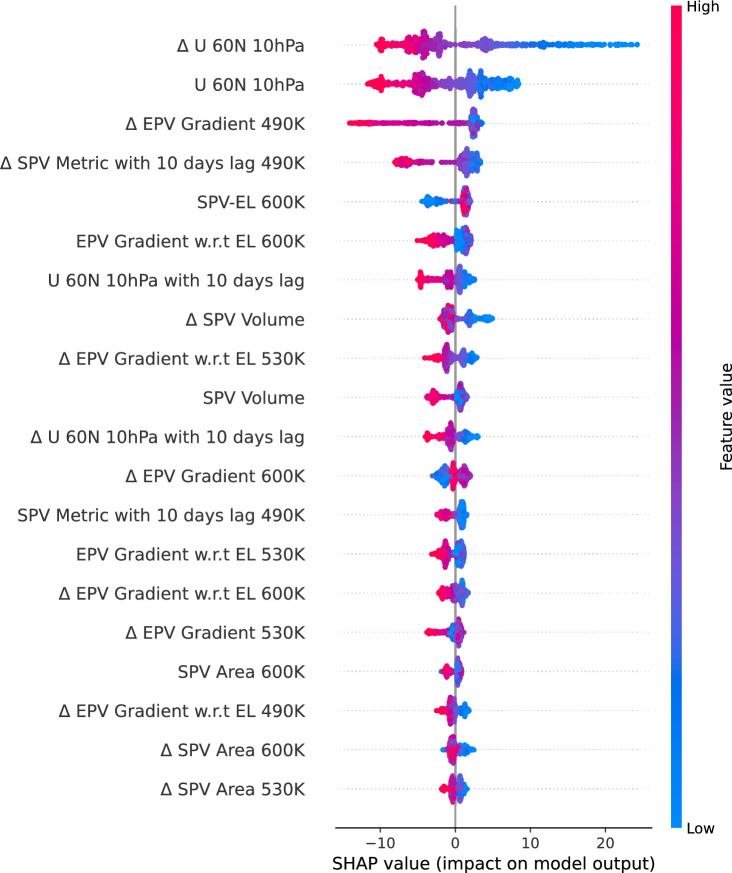
SHAP summary plot of XGBoost illustrating the contribution of different features to the model output. Features are ranked along the y-axis by their importance (mean SHAP value), with the most influential predictors appearing at the top. SHAP values are shown on the x-axis, which demonstrates the impact of a feature value on the model output. Each dot corresponds to the SHAP value of a feature for a single datapoint. Dots with positive SHAP values indicate a positive impact on the prediction, i.e., an increase in the estimated ozone, while dots with negative SHAP values indicate a negative contribution to the model output. The color gradient reflects the feature’s actual value, with blue denoting lower values and red denoting higher values. Here, $\Delta$ denotes the climatological anomaly of the respective feature.

**Fig. 8:**
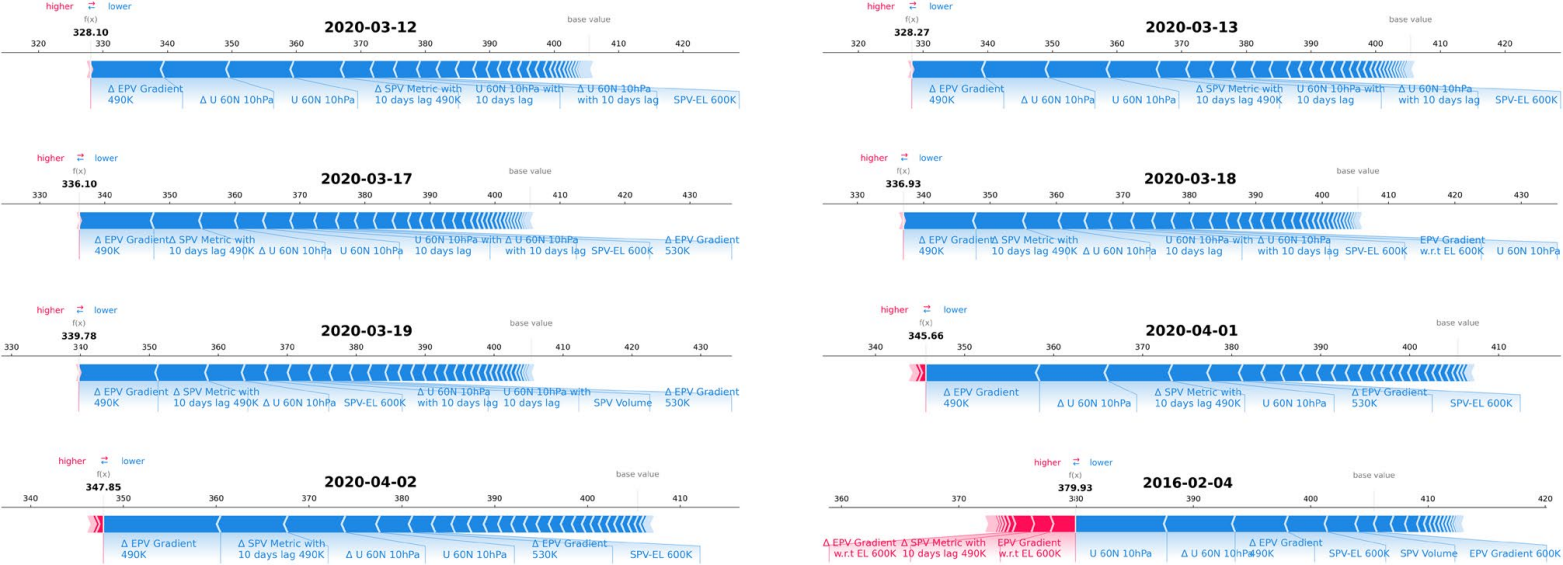
SHAP force plots illustrating the explainations of ML predictions on specific days i.e., March 12, 13, 17, 18, 19, April 1 and April 2, 2020 when ozone hole was recorded. In addition, February 4, 2016, chosen randomly for comparison outside extreme conditions. $\Delta$ represents the climatological anomaly of each feature.

### Explainability of the algorithm

Despite their remarkable performance, machine learning models are often regarded as black boxes. However, for scientific applications, ensuring the reliability of ML models is crucial. To provide proper explainability for our algorithm, we employed SHAP (SHapley Additive exPlanations)^38^, which allows us to quantify and visualize how each feature contributes to the predictions. This approach also enables us to verify the scientific rationale behind the weighting of specific features. Figure 7 presents the SHAP diagram for the top 20 features corresponding to the best performing ML model in our study (i.e., XGBoost). The x-axis represents the SHAP values, indicating the influence of each feature on the model’s predictions. Positive SHAP values signify that a feature increases the predicted ozone values, while negative values indicate a decrease in prediction. The color gradient, ranging from blue (low feature value) to red (high feature value), illustrates how different feature values impact the model’s predicted ozone values.

The top two features in the SHAP analysis, namely zonal mean zonal wind (U 60N 10hPa) and the climatological anomaly ($\Delta$ U 60N 10hPa), indicate that higher zonal wind values correspond to lower predicted ozone values and vice versa (Fig. 7). This relationship is also reflected in Fig. 1, which shows a negative correlation between U 60N 10hPa and ozone. In particular, the most extreme values of ($\Delta$) U 60N 10hPa and U 60N 10hPa contributed approximately between -10 to 21 DU and -10.1 to 10, respectively (Fig. 7). Moreover, higher values of $\Delta$ EPV gradient at 490K, $\Delta$ SPV Metric with 10 days lag, EPV gradient with respect to (w.r.t) Equivalent Latitude (EL) 600K, U 60N 10hPa with 10 days lag and $\Delta$ SPV Volume also negatively impact the predictions, while lower values lead to an increase (Fig. 7). These features represent either the strength or size of the SPV (details are described in Table 2). These features also exhibit a negative correlation with ozone (Fig. 1), indicating that an increase in SPV strength of the vortex edge or size results in a decrease in ozone values. The large vortex area and stronger vortex edge create an isolated and robust barrier that traps cold air, leading to the formation of PSCs and increasing chemical ozone depletion^9,21,39^. In contrast, a shrunken and weaker edge allows ozone rich air to mix in, thereby mitigating ozone losses if PSCs are formed^9,10^. In contrast, SPV-EL, which represents the position of the SPV edge, positively impacts the model predictions at higher values, while lower values decrease them. This relationship is consistent with the (Fig. 1) where a positive correlation between SPV-EL and ozone was observed.

Studies show that the vortex edge plays a critical role, as it provides greater stability to the vortex and prevents it from being easily disrupted by upward propagating tropospheric waves^21,39^. A somewhat similar tendency is seen in the SHAP analysis, where features related to the vortex edge show relatively higher influence compared to those describing its area. These explanations and scientific evidence, which include the strengthening of SPV dynamics and ozone loss, begin with a link to unusually low wave activity from the troposphere, resulting in a relatively undisturbed SPV. Moreover, at the same time, downward wave coupling enhances dynamical cooling and further strengthens the SPV. This undisturbed and intensified SPV creates a strong transport barrier, leading to extremely low temperatures that favor the formation of PSCs. In the presence of sunlight during late winter and early spring (FMA), ozone loss accelerates. Hence, the ESV were conducive to substantial ozone loss^21^.

In addition to the explainability of predicting extreme ozone loss events, we have used the force plot to investigate individual cases in Fig. 8. The SHAP force plot shows how individual features push the model prediction higher (red) or lower (blue) relative to the base value on a particular day. The base value represents the average training output or the algorithm’s average ozone prediction. Figure 8 highlights the days of March 12, 13, 17, 18, 19, April 1, and April 2, 2020, which correspond to ozone hole days, while February 4, 2016, is randomly chosen for comparison to observe the algorithm’s behaviour outside extreme cases. On ozone hole days, the feature representing the strength or size of the SPV consistently pushes the ozone prediction to lower values (shown in blue). This aligns with scientific understanding, as ozone loss or the reported ozone hole results from ESV^8,21^. Moreover, on February 4, 2016 (a normal day), some features influenced the prediction in both directions, either increasing or decreasing the ozone value.

## Discussion and conclusion

In this study, we have developed a novel approach to predict ozone using the morphological and dynamical properties of the SPV. To optimize the algorithm, we tested three ML models, namely XGBoost, DT, and MLP. Among these, XGBoost was identified as the best performing model, achieving the highest ($R^2$) of 0.80, a correlation of 0.91, and the RMSE of 16.78 and MAE of 13.01. Our algorithm (i.e., XGBoost) effectively represents annual standardized anomaly variations in alignment with observed ozone values. It is specifically designed to capture Arctic ozone variations, which are directly influenced by SPV dynamics. The development of this algorithm is particularly relevant in this decade, as three ESV events associated with ozone loss during the FMA months have been reported in recent decades^20–22^. Notably, 2020 showed the lowest ever TOC in the Arctic, marking the first reported ozone hole over this region^8,21^. Some studies suggest that such events could persist or even worsen by the end of the century if GHGs concentrations continue to rise steeply^9^. Additionally, Nilsen et al.^7^ reported significant negative trends at several stations for 20-year time windows ending after 2019, based on Arctic ozonesonde data spanning 1994–2022, highlighting the importance of continued monitoring and predictive modeling.

Considering the relationship between ESV and ozone loss, we utilized various morphological and dynamical properties of the SPV to predict ozone levels. Our algorithm successfully depicted the time series pattern of the record low ozone levels observed in 2020^8,21^, albeit with an overestimation of approximately 20 DU. It is important to note that our training dataset contained only two ESV (1997 and 2011) associated ozone loss events. Additionally, our algorithm does not incorporate any chemical processes as features, which may contribute to certain prediction limitations. For instance, the model underestimates high ozone values in 2024 by approximately 50 DU, due to its highest level ever over the Arctic in March 2024^37^. Our algorithm lacks any training data for such conditions. Despite these limitations, the algorithm effectively replicated ozone variations in 2016, 2017, 2018, 2021, 2022, and 2023. Therefore, the algorithm is performing relatively well in lower ozone years while in case of high ozone values, it may show slight underestimation. Furthermore, the algorithm provides a scientifically sound explanation of ozone variability^20–22^ through SHAP analysis, which quantifies feature importance and interprets the algorithm’s predictions. SHAP analysis is based on the assumption that the feature variables are independent^40^. As this is not necessarily true, it represents a limitation of our study. A recently developed approach, the SHAPR method^41^, accounts for the dependencies between the features. Incorporating SHAPR into future work could provide a more robust interpretation of the ML model.

The future scope of this study includes improving the model’s accuracy in quantitatively predicting both high and low ozone values. The algorithm can also be used as a fast algorithm to be used in CMIP models which do not have a chemistry package to predict ozone. Moreover, incorporating chemical data could be a valuable enhancement, as it may improve the algorithm’s performance. In particular, assuming that ODSs continue their slow decline towards the end of the century^10^, it can be considered as a limitation of the study. However, at present, the availability of observational chemical process data over the Arctic is dispersed and limited, making it difficult for training an ML model effectively. Nevertheless, this study presents an important finding by explicitly utilizing the physics of the SPV to predict, to some extent, chemical ozone loss with $R^2$ score of 0.80 and 0.91 correlation with observation. Future research could explore extending this algorithm to predict the spatial distribution of ozone over the Arctic, further enhancing its applicability and reliability. One may also explore applying the proposed ML approach to the Antarctic, where data characteristics differ from the Arctic.

## Methods

### Data processing

The TOC was extracted from NASA’s Ozone Watch mission^42^. The algorithm input features were calculated using the ERA5 reanalysis dataset^43^ and characterize the dynamical and morphological properties of the Arctic SPV.

The selected features include SPV area, Ertel’s Potential Vorticity (EPV) gradient, EPV gradient with respect to (w.r.t) Equivalent Latitude (EL), SPV-EL, and SPV center value, all of which were used at the three isentropic levels 490K, 530K, and 600K. Additionally, daily zonal mean zonal wind at $60^\circ N$ at 10 hPa and its 10-day lagged values were included, along with SPV volume and SPV metrics with a 10-day lag. The 10-day lag is defined as the arithmetic mean of the last 10 days of data. The units and descriptions of all features are provided in Table 2. In addition to these features, the ML algorithm also incorporated the daily climatological anomaly ($\Delta$) of all variables, which has proved to improve the results^26,28^.

**Table 2:** Description of all features used to develop the ML algorithm.

Features (Unit)	Descriptions
SPV Area (Million km$^2$)	SPV Area refers to the size of the SPV at a specific isentropic level. It is determined using a constant contour of EPV value. These constant values (490 K, 530 K, and 600 K, corresponding to 41.36, 62.19, and 113.16, respectively) are based on the climatological mean of the daily calculated vortex edge^44^ from the 1979 to the 2023 NH winter (November to April)^39,45,56^.
EPV Gradient (EPVU)	EPV gradient quantifies the strength of the vortex edge based on EPV. It is defined as the maximum EPV gradient from the equator to the pole ($30^\circ$ and $80^\circ$ latitude). The unit of EPV is expressed in EPVU ($10^6 \, \text {m}^2 \, \text {K} \, \text {s}^{-1} \, \text {kg}^{-1}$).
EPV Gradient w.r.t EL (EPVU/°N)	EPV Gradient w.r.t EL also quantifies the strength of the vortex edge based on EPV but as a function of EL. Similar to the EPV gradient, it is defined as the maximum EPV gradient from the equator to the pole within the latitude range $30^\circ$ and $80^\circ$.
SPV-EL (°N)	SPV-EL represent the position of the SPV edge. It is defined using the EPV gradient w.r.t EL, within the latitude range $30^\circ$ and $80^\circ$
SPV Center Value (EPVU)	SPV center is defined as the location where the maximum value of EPV is identified within the vortex.
SPV Volume (Million km³)	SPV volume is calculated based on the vortex area, extending from the lower to the upper stratosphere.
SPV Metric with 10 days lag ((Million km² EPVU)/°N)	SPV metric is defined by the multiplication of SPV area and EPV Gradient w.r.t EL. It is a good proxy to define the strength of the SPV. We use the SPV metric with a 10-day lag, meaning the average of the last 10 days of the SPV metric to define the current day’s strength of the SPV.
U 60N 10hPa (m/s)	Zonal mean zonal wind at 10 hPa and $60^\circ N$ latitude.
U 60N 10hPa with 10 days lag (m/s)	Zonal mean zonal wind at 10 hPa and $60^\circ N$ latitude, considering a 10-day lag, meaning the average of the last 10 days of zonal wind to define the current day’s zonal wind.

### Feature selection

Feature selection was based on the physical relationship between SPV dynamics and Arctic ozone^13,16,18–22^. Scatter plots showing the correlation of these features with TOC are given in Fig. 1. Negative correlations of SPV area, EPV gradients, SPV center value, SPV volume, and zonal wind at 490K, 530K, and 600K, indicate that stronger and more stable SPV conditions are associated with lower ozone values. Particularly, zonal wind shows -0.62 correlation with TOC, highlighting its crucial role in modulating stratospheric dynamics and ozone variability^21^. Lags of 10 days in zonal wind and SPV metrics maintain their correlation strengths, suggesting a time dependent persistence of the SPV influence on ozone. SPV-EL represents the SPV edge position. Greater SPV-EL values represent a vortex edge nearer to the pole, thus smaller size of the vortex. Positive correlation of SPV-EL and TOC indicate that smaller SPV-EL corresponds to lower ozone, i.e, a larger vortex will support ozone loss. Physical relation of SPV dynamics and Arctic ozone confirms that the strength and morphology of the stratospheric polar vortex play a critical role in determining ozone concentration variability.

### Algorithm descriptions

Our algorithm is designed to predict the ozone without solving the chemical rate partial differential equations^46^. TOC averaged over the polar cap ($> 63^\circ$ N) is used as ozone. To predict the TOC over the polar cap, an ML algorithm is employed, considering only features that provide information on the morphological and dynamical properties of the SPV. Three ML models have been considered to find the optimal algorithm for predicting ozone, namely XGBoost^34^, Decision Tree (DT)^35^, and Multilayer Perceptron (MLP)^36^. These ML models were trained using a daily dataset spanning from 1980 to 2024. The entire dataset was split into training (1980–2015) and testing (2016–2024) periods. The training dataset was selected to cover at least two ESV events (1996-97, 2010-11), which are associated with late winter and early spring ozone loss over the Arctic^20–22^. One of the aims of the model is to predict the lowest recorded ozone values (2019-20) over the Arctic^8,21^ during the testing period. Two years of TOC data (1995 and 1996) are missing due to the loss of the Tropical Rainfall Measuring Mission (TRMM) satellite^42^; therefore, these years were excluded from the training dataset. We excluded the missing years when calculating climatological anomalies for all variables. It is important to note that the ML algorithm considers only data from the late winter and early spring months (i.e., FMA).

For the scientific interpretation of the ML algorithm, we used SHAP (SHapley Additive exPlanations), which provides SHAP values to quantify the contribution of different features in the algorithm^38^, particularly the TreeSHAP method has been used^47^. Several recent studies^48–55^ have employed the SHAP method in atmospheric and climate science to interpret the explainability of ML algorithm. The algorithm’s performance was evaluated against observations using the correlation coefficient (R), $R^2$ (coefficient of determination), root mean square error (RMSE), and mean absolute error (MAE). To assess the uncertainty of the ML algorithm, it was also tested with ten different random seed values, and the results were reported accordingly.

## Supplementary Information

Supplementary Information.

## Data Availability

The NASA polar cap ozone values can be accessed at https://ozonewatch.gsfc.nasa.gov/NH.html. ERA5 reanalysis data are available from the Copernicus Climate Data Store at https://cds.climate.copernicus.eu/datasets/reanalysis-era5-pressure-levels?tab=overview.
