# Supplementary material for: A novel approach to predict the arctic stratospheric ozone from stratospheric polar vortex dynamics using explainable machine learning
Source: Sci Rep. 2025 Oct 20;15:36473. doi: 10.1038/s41598-025-24379-9 (PMC12537872; doi:10.1038/s41598-025-24379-9)
Supplement: Supplementary file 1 — Supplementary Information. [file 41598_2025_24379_MOESM1_ESM.pdf]

Supplementary Information

Comparisons of Different ML Models

Comparisons of different ML models based on one random seeding value using various parameters such as the annual interannual variability of ozone standardized anomalies, the frequency distribution of observed and predicted ozone values, the scatter plot of observed and predicted ozone values, and the time series of observed and predicted ozone levels for the February, March, and April months from 2016 to 2024 are provided here, which is the testing period of ML models. The relevant figures are Figures 1, 2, 3, and 4.

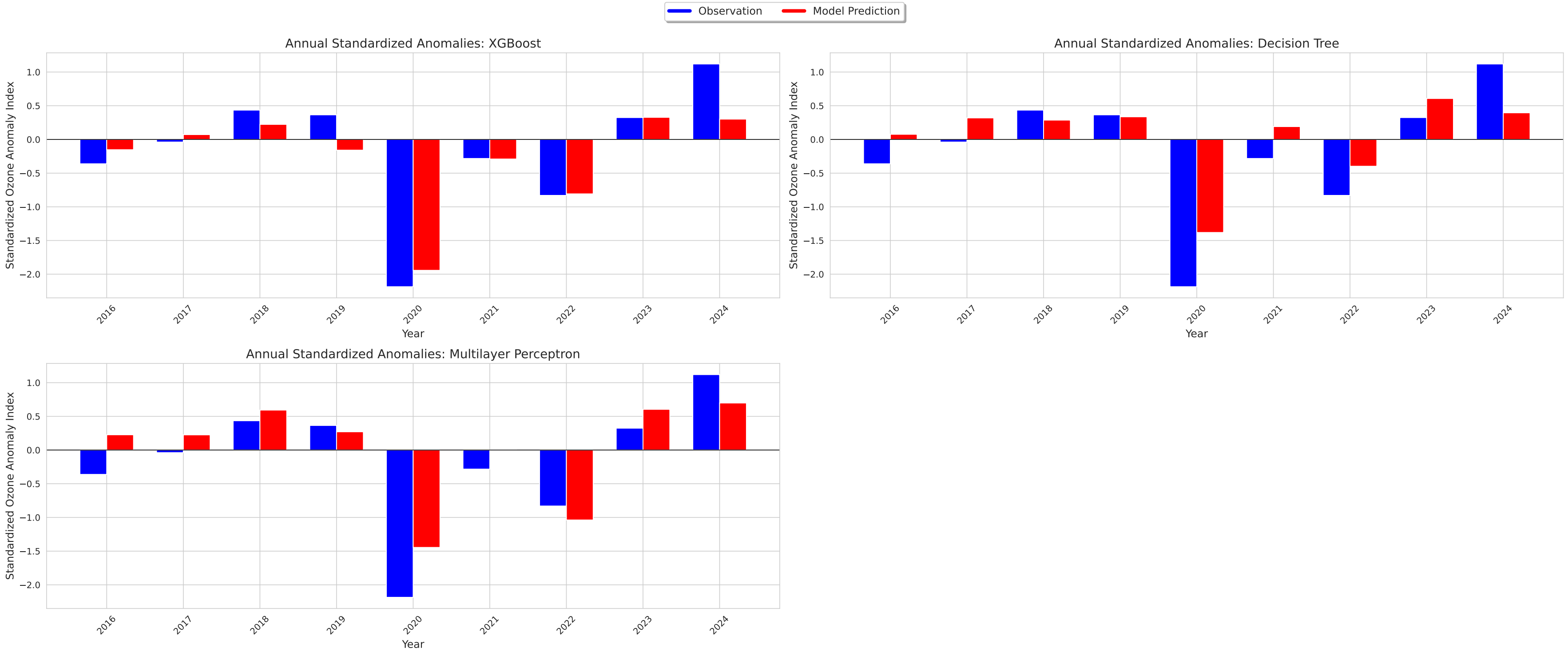

Figure 1: Annual standardized anomalies variation of observation and XGBoost, Decision Tree, and Multilayer Perceptron algorithm.

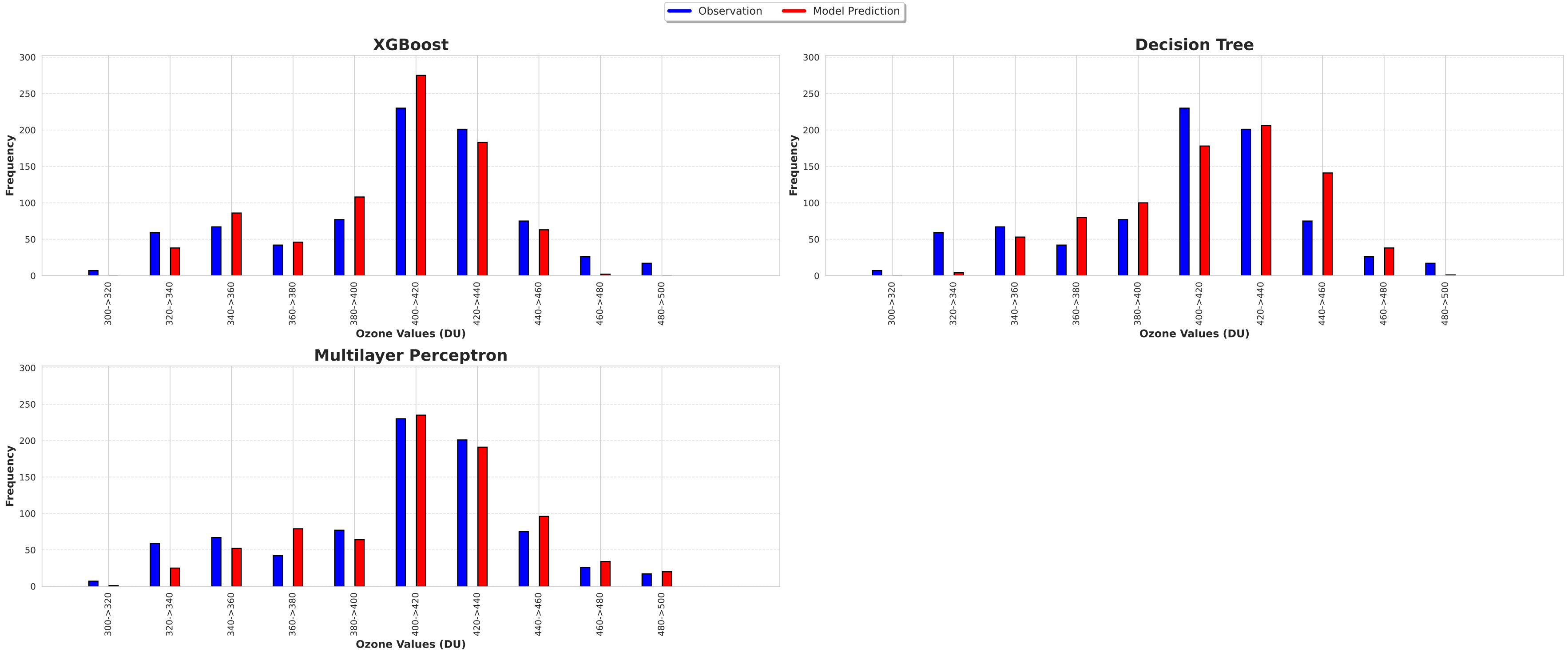

Figure 2: Ozone distribution of observation and XGBoost, Decision Tree, and Multilayer Perceptron algorithm.

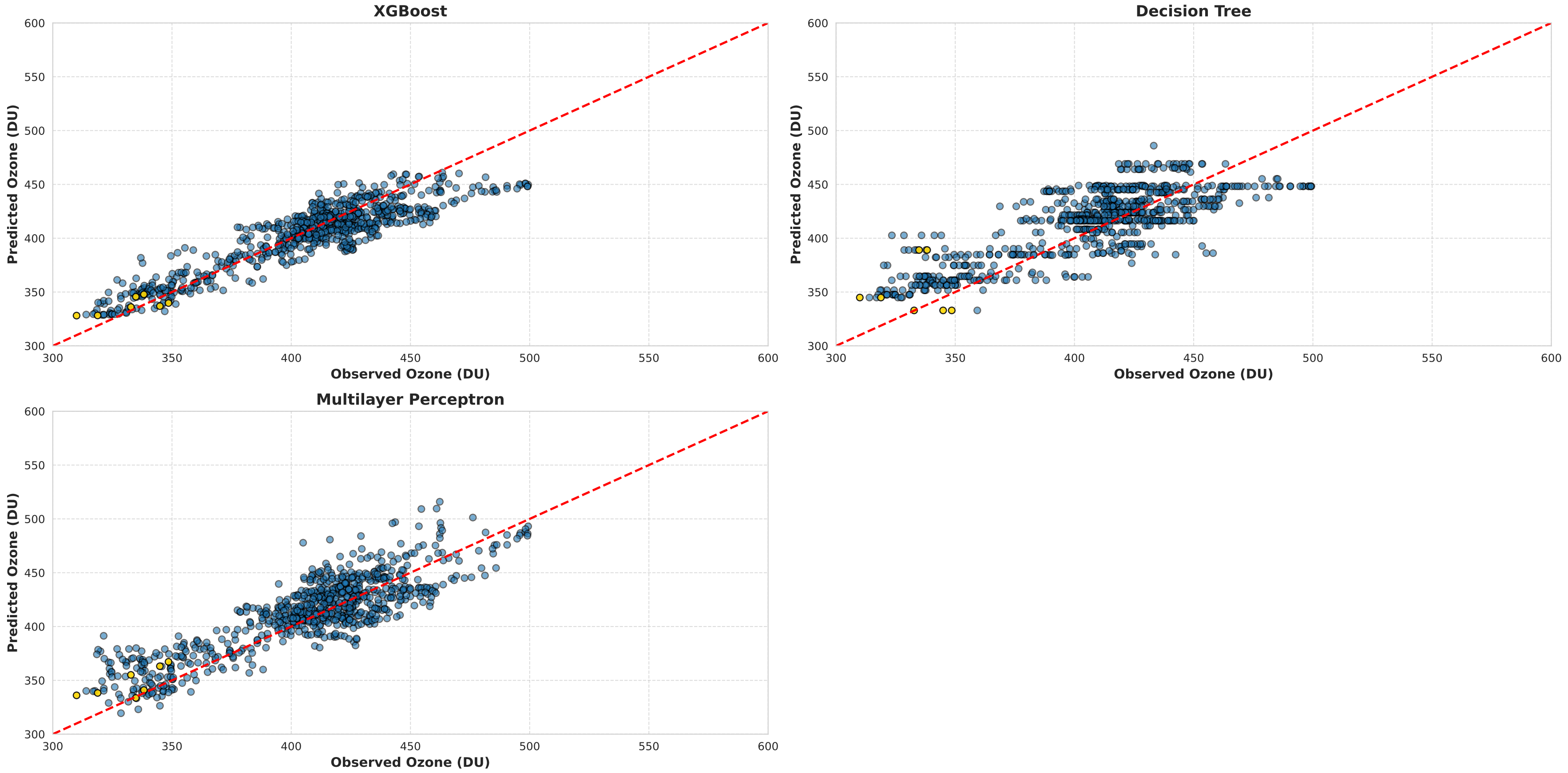

Figure 3: Observed and XGBoost, Decision Tree, and Multilayer Perceptron predicted ozone with 0.91, 0.82, and 0.85 correlation coefficient, respectively. The yellow dotted lines represent the dates when the ozone hole was recorded (2020-03-12, 2020-03-13, 2020-03-17, 2020-03-18, 2020-03-19, 2020-04-01, 2020-04-02).

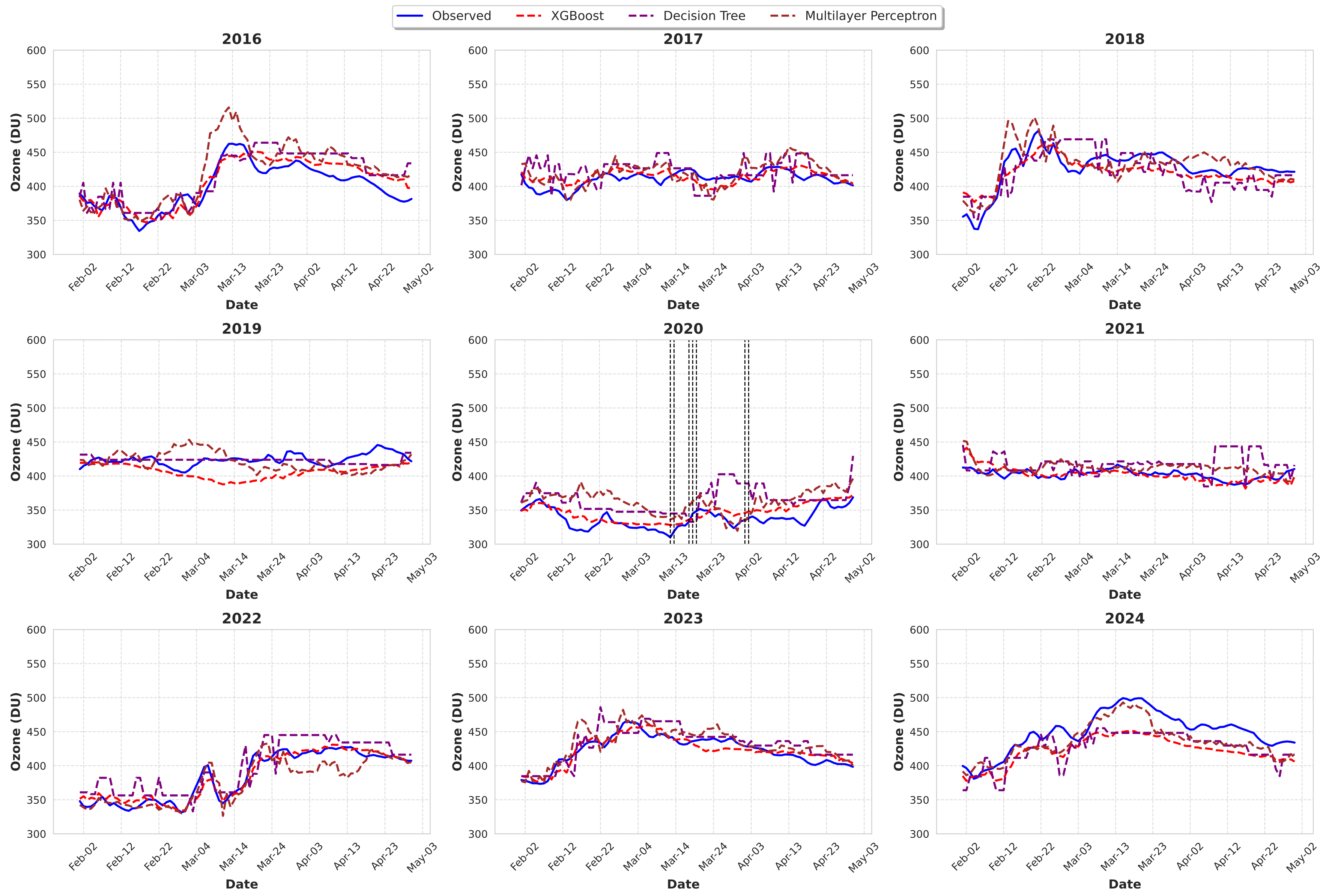

Figure 4: Time series of Observed and XGBoost, Decision Tree, and Multilayer Perceptron predicted ozone during FMA for the testing period (2016-2024). Black dotted vertical line represents dates when the ozone hole was recorded in 2020.
